# Study on the treatment of osteoarthritis by acupuncture combined with traditional Chinese medicine based on pathophysiological mechanism: A review

**DOI:** 10.1097/MD.0000000000037483

**Published:** 2024-04-05

**Authors:** Biao Qi, Zeyu Wang, Ying Cao, Haishen Zhao

**Affiliations:** a Shenzhen Baoan District Shiyan People’s Hospital, Shenzhen, China; b Shenzhen Pingshan District Hospital of Traditional Chinese Medicine, Shenzhen, China; c Community Health Service Center of Nanhui New Town, Shanghai, China

**Keywords:** acupuncture, acupuncture combined with TCM, OA, pathophysiological, TCM

## Abstract

Osteoarthritis (OA) is a major contributor to disability and social costs in the elderly. As the population ages and becomes increasingly obese, the incidence of the disease is higher than in previous decades. In recent years, important progress has been made in the causes and pathogenesis of OA pain. Modern medical treatment modalities mainly include the specific situation of the patient and focus on the core treatment, including self-management and education, exercise, and related weight loss. As an important part of complementary and alternative medicine, TCM has remarkable curative effect, clinical safety, and diversity of treatment methods in the treatment of OA. Traditional Chinese Medicine treatment of OA has attracted worldwide attention. Therefore, this article will study the pathophysiological mechanism of OA based on modern medicine, and explore the treatment of OA by acupuncture combined with Chinese Medicine.

## 1. Introduction

Osteoarthritis (OA) is a common and disabling disease and is the most prevalent musculoskeletal disorder.^[1,2]^ OA is one of the leading causes of disability in the United States, with more than 22.7 million people experiencing activity limitations due to arthritis.^[3]^ Only depression and alcoholism cause more disabling losses than OA.^[4]^ OA has become more prevalent with the combined effects of an aging global population and increasing obesity rates, as well as an increase in the number of joint injuries.

OA is a complex chronic disease, often compounded by the coexistence of multiple diseases.^[5]^ Typical management approaches are palliative and passive, rather than focusing on shared decision-making or coordinated, proactive and preventive behavior. However, considering the increasing burden of OA on individuals and society, the management approach should be changed to provide individualized care based on the specific needs of patients. These can be well achieved through complementary and alternative medicine.

In view of the recent changes in the concept of OA pathogenesis and pain etiology. Therefore, based on the latest progress in the pathophysiology of OA, this paper will explore the treatment of OA with the combination of TCM and acupuncture.

## 2. Epidemiology of OA

The relationship between old age, obesity, and increased incidence of knee OA is well known. However, a study by Wallace et al^[6]^ showed that these major risk factors are not sufficient to explain the exponential increase in the incidence of knee OA. They found that the incidence of knee OA has doubled since the mid-20th century. Even after controlling for age and body mass index. This finding suggests that interactions with the modern environment may play a pathogenic role in the development of OA. These authors hypothesized that reduced physical activity may be an important factor. Because this leads to chronic underloading of the joints, reduced proteoglycan content, weakened muscles, and the inability to protect and stabilize the joints.

Clinically, knee joint is the most common site of OA, followed by hand and hip.^[7]^ A systematic review^[8]^ showed that the incidence of OA reported in various studies depended on the definition of OA used as well as the age category. A Swedish registry data study^[7]^ counted the proportion of people over 45 years of age with any form of OA diagnosed by a physician, which was 26.6% in the Skane region of southern Sweden in 2012. Of 531,254 prevalent cases, 141,313 were polyarticular OA cases. It is estimated that by 2032, the proportion of people over 45 years of age with physician-diagnosed OA will increase from 26.6% to 29.5%. The proportion of any part will increase, from 13.8% to 15.7% for the knee and from 5.8% to 6.9% for the hip.^[7]^

Data from the Spanish and UK GP registries were used to report the incidence of OA in the general population^[9]^ and showed that the effect of age on the individual risk of OA in the hip, hand, and knee in women followed a similar pattern, with the risk of knee and hand increasing rapidly between the ages of 50 and 75. The incidence of women is much higher than that of men. These 2 studies have shown that the peak incidence of OA generally occurs around the age of 75.^[1]^

Two papers by Davis et al demonstrate that certain patients are at risk for accelerated OA and that these patients are more likely to undergo knee replacement. These patients also reported more frequent pain, more frequent knee swelling, and a greater likelihood of activity restriction due to pain.^[10]^ Patients with accelerated OA compared with those without accelerated OA had a specific set of symptoms at the 1-year index examination. These patients feel worse when they lie down, even more pain in their knees, and more pain when they walk. Accelerated OA is not benign, as these patients have 25 times the chance of having a knee replacement within 9 years compared to patients with radiographic OA of the knee without rapid progression.^[11]^

## 3. Genetics of OA

OA is a polygenic disease, and researchers conducting OA genomic studies have focused on typically diagnosed cases of age-related disease, which begins in the fifth decade of life.^[12]^ In a phenotype-wide association study using the UK Biological Database, heterozygotes for the Z allele of SERPINA1 (rs28929474) were associated with multiple musculoskeletal phenotypes, including reduced risk of OA but increased risk of osteoporosis and reduced bone mineral mass.^[13]^ AAT inhibited osteoclast formation and bone resorption induced by RANKL. Recently, neutrophil elastase, for which AAT is an inhibitor, has been shown to fully activate MMP13, the major collagenase in OA that promotes the irreversible destruction of cartilage collagen.^[14]^

GLIS3 is a transcriptional regulator that regulates hedgehog signaling, a pathway involved in limb development and OA severity.^[15]^ However, the factor itself is strongly associated with neonatal diabetes and congenital hypothyroidism, although single nucleotide polymorphisms around GLIS3 are associated with OA susceptibility.^[16]^ This year, Zhang et al^[17]^ confirmed similar OA associations in Chinese population (rsl0116772, rs7045410, and rs7032713).

Most of the existing OA genomics data come from cartilage tissue. There have been several genomics studies this year that have examined other tissues of the joint and investigated the relationships between the joint tissues. Tuerlings et al^[18]^ used RNA sequencing to study subchondral bone, highlighting differentially expressed genes between paired preserved and diseased regions in 18 patients with KOA and 6 patients with HOA. Comparison with articular cartilage revealed common altered genes between joint sites, including the OA susceptibility genes fill and CHADL, which are candidates for future studies in in vivo OA models. In a multi-tissue holographic study, Steinberg et al^[19]^ performed transcriptomic and proteomic studies on cartilage and synovial tissues from 115 OA patients and exome genotyping in peripheral blood. The authors found 1891 cis-eQTLs and 38 cis-protein-eQTLs for genes. The authors demonstrate the utility of this molecular map in identifying the effector genes of noncoding Genome-Wide Association Studies guided variants. The authors examined the enrichment of 148 upregulated proteins in damaged cartilage in a library of gene expression changes in vitro drug therapy, thereby computationally screening for opportunities for drug reuse. Drugs with the highest concentrations, such as IB-MECA, vascular endothelial growth factor-2-Jimei inhibitor-IV, and nominicotinamide, may reduce the expression of the up-regulated characteristics of OA-damaged cartilage.

Recently, there are few studies on OA which is all DNA methylation. However, Kashin-Beck’s study is an exception.^[20]^ Smeriglio et al^[21]^ showed that the accumulation of 5-hydroxymethylpyrimidine (5 hmC) in DMM-induced OA in mice coincided with changes in gene expression, building on the group’s previous work on the implicit role of 5 hmC in OA.^[22]^ The oxidation of 5-methylcytosine to 5hmC is the first step in DNA demethylation and can be mediated by the methylcytosine dioxygenase TET1.

Yang et al^[23]^ found that oxidative stress in OA also increased with age, and they determined the expression changes of 550 circulating RNAs after 5 days of H2O2 treatment of chondrocytes to induce oxidative stress. The most upregulated was circRSUl, and experimental manipulation of its levels altered the balance of chondrocyte gene expression toward catabolism, with increased metalloproteinase expression and decreased matrix gene expression. The authors demonstrated that circRSUl had ceRNA activity on several miRNAs, but it was miR-93-5p that was evident. Notably, circRSUl intra-articular administration induced OA in mice with the same severity as DMM surgery, whereas mutation of the miR-93-5p binding site in circRNA completely abolished this effect. Finally, they established a mechanism by which miR-93-5p targets MAP3K8, thereby inhibiting extracellular signal-regulated kinase and NF-κB signaling. This is reversed by experimental or age-related up-regulation of circRSUl and a corresponding decrease in available miR-93-5p.

## 4. Pathophysiological mechanisms of OA

OA is a total joint disease involving changes in hyaline key cartilage, subchondral bone, ligaments, joint capsule, synovium, and periarticular muscles. The disease is an active, dynamic change that arises from an imbalance between the repair and destruction of joint tissue, rather than a passive degenerative disease or a so-called wear and tear disease, as it is often called.^[24]^

During the pathogenesis of OA, the cartilage composition changes and the cartilage loses its integrity.^[25]^ Changes in composition alter the material properties of cartilage, increasing its susceptibility to destruction by physical forces. Initially, erosion occurs only on the surface, and later the cartilage gap becomes deeper and deeper, and the calcified cartilage area expands. In order to repair, hyperplastic chondrocytes show greater synthetic activity, but in the process, matrix degradation products and proinflammatory mediators are produced, thereby deactivating chondrocytes. It also acts on the adjacent synovium to stimulate proliferation and pro-inflammatory responses. Proinflammatory products are also released by proliferating synovial cells; this process is accompanied by tissue hypertrophy and increased vascularity. In the subchondral bone, bone turnover is increased and blood vessels invade from the subchondral bone through the tendon scar and into the cartilage. This bone remodeling and repair is also associated with the development of bone marrow lesions in the subchondral bone. Hyperostosis formed at the joint edge by reactivation of endochondral ossification.^[26]^

OA is often described as a heterogeneous disease with a wide variety of potential pathogenic pathways and similar outcomes leading to joint destruction.^[27]^ Each of the common OA risk factors may lead to a different mechanistic pathway to OA. The most disabling symptom in OA patients is pain. Pain is a major driver of clinical decision making and health service use. Patients with OA also frequently experience joint instability, swelling, muscle weakness, fatigue, and psychological distress associated with pain.

The pain of knee OA is usually intermittent, mainly weight-bearing pain. Usually, intermittent pain can be predicted, but when pain becomes more severe, more frequent, or unpredictable, patients often classify it as unacceptable pain.^[28]^

In addition to peripheral pain mechanisms, hyperalgesia due to neuropathic or central pain mechanisms appears to be present in most patients with synovitis.^[29–31]^ Neuropathic pain arises from structural changes in the innervation of joints, or from neurological changes in the peripheral nervous system or spinal cord.

A meta-analysis of quantitative sensory testing to objectively measure pain sensitivity in OA patients^[32]^ showed that there were differences in pain pressure threshold, time summation, flexor withdrawal response and conditioned pain regulation between patients and healthy controls.

Based on a self-reported neuropathic pain screening questionnaire, a meta-analysis^[33]^ showed that 23% of OA patients may have some form of neuropathic pain.

## 5. Diagnosis of OA

Clinical diagnosis is the criterion for the diagnosis of OA, based on symptoms (pain, transient morning stiffness, and functional limitations) and a brief physical examination (creaking, limited or painful movement, tender joints, and hyperostosis). Clinically recommend appropriate diagnostic criteria, For example, the American College of Rheumatology or the European League Against Rheumatism for knee OA.^[34,35]^ However, the need to use early OA diagnostic criteria is emphasized in order to identify patients at an early stage of the disease and initiate critical treatment from the earliest symptoms.^[36]^ The initial evaluation should include a complete history and physical examination to determine the impact of OA on function, quality of life, mood, social engagement and relationships, occupation, leisure activities, and sleep,^[37]^ and to identify associated comorbidities. This holistic assessment promotes cooperation and collaborative care between patients and professionals, thereby facilitating shared decision-making and ultimately improving treatment outcomes.^[38]^

## 6. Etiology and pathogenesis of OA

OA belongs to the category of “arthralgia syndrome” and “bone arthralgia” in Traditional Chinese Medicine (TCM). The discussion of “arthralgia syndrome” and “bone arthralgia” was first found in Huangdi Neijing. There is a special chapter on arthralgia syndrome in Huangdi Neijing Suwen, which has a clear understanding of the etiology, pathogenesis and syndrome differentiation of arthralgia syndrome. Su Wen Bi Lun Pian says: “The so-called arthralgia is named for its time, and it is heavily influenced by wind, cold, and dampness.” It is pointed out for the first time that wind, cold, dampness and other exogenous pathogens are the key causes of Bi syndrome. It is also said in Su Wen Bi Lun Pian: “The five Zang organs are all in harmony, and those who are ill for a long time and do not go away should give up their harmony.” Therefore the bone arthralgia unceasingly, again feels in the evil, inside gives up in the kidney. The skin arthralgia is unceasingly caused by pathogenic factors, and the internal arthralgia is caused by the lung. It is emphasized that the occurrence of arthralgia syndrome is not only related to the invasion of exogenous pathogens, but also closely related to the normal functions of viscera, nutrient and defensive qi and blood, meridians and blood vessels. The dysfunction of viscera, the disharmony of nutrient and defensive qi, the deficiency of qi and blood, and the obstruction of meridians and blood vessels lead to the occurrence of various arthralgia syndromes.

Modern physicians have further improved the theory of etiology and pathogenesis of OA. Professor Zhu Liangchun believes that the etiology of OA can be divided into internal cause and external cause, the internal cause is deficiency of kidney-yang, disharmony of qi and blood, and dystrophy of tendons and marrow, and the external cause is external pathogenic factors such as wind, cold, blood stasis, heat and dampness. Exogenous pathogens invade and stay in the meridians and collaterals, blocking the circulation of qi and blood, and the deficiency of liver and kidney can not nourish the limbs and bones to resist exogenous pathogens, resulting in OA disease.^[39]^ Professor Yan Xiaoping believes that the occurrence of OA is mostly due to deficiency of liver and kidney, wind, cold, dampness, heat and other pathogenic factors invading the body and blocking the meridians.^[40]^

Most ancient and modern physicians believe that the pathogenesis of this disease is deficiency of vital qi and excess of pathogenic factors. Deficiency of vital qi is caused by deficiency of liver and kidney, deficiency of qi and blood, weakness of spleen and stomach, Yang deficiency and cold coagulation, etc. Pathogenic excess is caused by external force, phlegm and blood stasis, dampness-heat stagnation or exogenous pathogenic factors leading to pain. It is difficult to absolutely distinguish excess of pathogenic factors from deficiency of vital qi.^[41]^

## 7. Mechanism of TCM in the treatment of OA

In the pathogenesis of OA, IL-1 is the core inflammatory factor, which is produced by inflammatory response caused by external stimulation. Fan et al^[42]^ showed that Bushen Tongluo Prescription could reduce the destruction of OA and improve OA symptoms by inhibiting MMP-3/13 and ADAMTs-4 levels. Minlong et al^[43]^ found that Wufuyin Granule could significantly reduce the content of IL-1β and IL-6 in serum and synovial fluid, delay the loss of proteoglycan in cartilage, and promote the synthesis of proteoglycan in articular cartilage, thereby preventing and treating OA. Peipei et al^[44]^ found that Yunnan Baiyao can reduce the secretion of inflammatory mediators, inhibit inflammatory response, significantly increase the mechanical pain threshold, reduce the sensitivity of pain receptors, improve the neuropathic pain caused by OA and reduce joint and synovial injury by reducing the level of serum PGE2.

IL-1stimulates synovial cells to produce IL-6.^[45]^ IL-6 promotes the activation of nuclear factorκB (NF-κB). The expression of receptor activator of nuclear factor-κ B ligand, together with the potent pro-inflammatory factor TNF-α^[46]^ produced by macrophages, which participates in the bone resorption process by activating the NF-κB pathway, participates in osteoclast differentiation and affects bone resorption. Weihong et al^[47]^ found that Qutan Bushen Prescription could control the development of OA by inhibiting the expression levels of inflammatory factors IL-1, IL-6, and TNF-α in serum and synovial fluid of rats. Yang et al^[48]^ found that Bushen Yigan Huoxue Prescription could reduce the content of IL-1β and TNF-α in serum, reduce the production of MMP-3 and improve the degeneration of articular cartilage.

Endoplasmic reticulum is the most adaptable organelle in eukaryotic cells, which is an important place for protein synthesis, processing and modification, sorting and transport, and lipid metabolism. Endoplasmic reticulum stress is a self-protection mechanism in vivo, which is involved in apoptosis. It has been shown^[49]^ that endoplasmic reticulum stress is present in chondrocytes from OA patients. Jun et al^[50]^ found that Duhuo Jisheng Decoction inhibited the mRNA and protein expression of PERK, Bip, elF-2α, ATF-4, GADD153, Caspase-9 and Caspase-3 by regulating the PERK/Bip signaling pathway, thereby inhibiting chondrocyte apoptosis caused by endoplasmic reticulum stress. Lincan et al^[51]^ found that Duhuo Jisheng Decoction can inhibit joint inflammation, thereby down-regulating PEAK/Bip signaling pathway and reducing chondrocyte apoptosis.

TGF-β is a structure-related signaling molecule, which plays an important role in extracellular matrix formation, tissue differentiation, bone remodeling, immune regulation and so on. BMP is a cytokine secreted by osteoblasts, and BMP-2, 4 and 7 promote osteoblast differentiation.^[52]^ The TGF-β/BMPs signaling pathway is regulated by a variety of factors and transduced through the Smad/R-Samd complex or MAPK cascade.^[53]^ When stimulated by exogenous factors such as cartilage injury, TGF-β/BMPs signaling pathway is activated and participates in the course of OA through a series of signaling.^[52]^ Fan et al^[54]^ found that Bushen Tongluo Prescription regulates the secretion of TGF-β1, BMP-4 and BMP-7 by regulating the TGF-β/BMPs signaling pathway, protects damaged chondrocytes, indirectly inhibits the destruction of chondrocytes, alleviates the progress of the disease and improves the prognosis.

## 8. Treatment of OA with TCM

Chaolun et al^[55]^ designed a randomized controlled trial (RCT) to evaluate the efficacy of Biqi Capsule in the treatment of knee osteoarthritis (KOA) complicated with bone marrow edema (BME). In this study, 90 patients with KOA and BME were randomly divided into experimental group (Biqi capsule treatment, 45 cases) and control group (etoricoxib treatment, 45 cases). Western Ontario and McMaster Universities Osteoarthritis Index (Western Ontario and McMaster Universities osteoarthritis index, WOMAC score, visual analogue scale score, whole-organ magnetic resonance imaging score, WORMS), the proportion of BME volume, and the peripheral blood inflammatory index peripheral blood neutrophil lymphocyte ratio to lymphocyte monocyte ratio to evaluate the efficacy of Biqi capsule. In addition, the correlation between BME volume and pain was observed to support the results of the analysis. Researchers found that Biqi capsule can effectively reduce the pain and improve the knee function of patients with KOA combined with BME, and the effect is better than etoricoxib. The reason for this advantage is that Biqi capsule can reduce the volume of BME.

Daoqing et al^[56]^ observed the clinical efficacy of modified Juanbi Decoction in the treatment of OA with qi stagnation and blood stasis syndrome based on the method of supplementing qi, activating blood circulation and dredging collaterals. In this study, 60 patients with the diagnosis of qi stagnation and blood stasis syndrome of OA were randomly divided into 2 groups, the observation group and the control group, with 30 cases in each group. The observation group was treated with modified Juanbi Decoction, and the control group was treated with etoricoxib. VAS score, WOMAC score, clinical efficacy, TCM syndrome score, inflammatory factors in synovial fluid and safety indicators were compared between the 2 groups before treatment and after 2 weeks of treatment. The researchers found that the levels of TNF-α and IL-1β in synovial fluid in the observation group were lower than those in the control group, and the clinical efficacy of modified Juanbi Decoction in the treatment of OA patients with qi stagnation and blood stasis syndrome was better, which could improve joint pain by reducing intra-articular inflammatory reaction.

Hou et al^[57]^ designed RCT to observe the clinical efficacy of Zhitong Siwu Decoction in the treatment of patients with KOA after arthroscopy. In this study, 100 patients with KOA after arthroscopic surgery were randomly divided into control group and observation group, 50 cases in each group. The control group was treated with conventional therapy after operation, while the observation group was treated with Zhitong Siwu Decoction on the basis of conventional therapy. The total effective rate, swelling regression time, hospital stay, knee flexion, Lysholm knee score, visual analogue scale (VAS) score, TNF-α, IL-1β, C-reactive protein (CRP) levels, Western Ontario and McMaster University Osteoarthritis Index Scale (WOMAC) item scores were compared. Researchers have found that Zhitong Siwu Decoction can promote the improvement of knee function, swelling and pain, inflammatory factors and other indicators in patients with KOA after arthroscopy, and can significantly improve the therapeutic effect.

## 9. Acupuncture treatment of OA

Yan et al^[58]^ designed RCT to explore the clinical study of 3-way acupuncture therapy for OA patients based on toll-4 receptor (TLR4)/NF-κB inflammatory axis. In this study, 102 patients with OA were randomly divided into control group and study group, with 51 cases in each group. The control group was given conventional treatment, and the study group was given 3-way acupuncture treatment on the basis of the control group. All patients were treated for 4 weeks. The TCM syndrome score, pain degree, inflammatory factor level, TLR4/NF-κ and joint inflammation index were observed and compared before and after treatment in the 2 groups, and the clinical efficacy was observed and compared between the 2 groups. Conclusion Three-way acupuncture can effectively relieve the clinical symptoms of OA patients, reduce the degree of pain, reduce the level of inflammatory factors, inhibit the TLR4/NF-κB inflammatory axis, and reduce the joint inflammation index, which has significant curative effect and can be widely used in clinic.

Qi et al^[59]^ observed the clinical efficacy of warm needle moxibustion combined with needling in the treatment of OA of liver and kidney deficiency type. In this study, 80 patients with KOA of liver and kidney deficiency type were randomly divided into observation group and control group, with 40 cases in each group. The control group was treated with warm acupuncture and moxibustion at local points, while the observation group was treated with warm acupuncture and moxibustion at Xuehai, Liangqiu, Ganshu, and Shenshu points combined with acupuncture on the basis of the control group, twice a week (Monday and Thursday) for 8 weeks. The changes of TCM syndrome score, KOA function (Lequesne) index score, VAS and daily living ability evaluation (Barthel score) were observed before and after treatment. Serum TLR4 and NF-κB were detected by ELISA. The levels of NF-κB, TNF-α, IL-1β, total antioxidant capacity and lipid peroxide were measured and compared between the 2 groups. Researchers believe that warming acupuncture and moxibustion combined with acupuncture can significantly improve clinical symptoms, reduce serum inflammatory factors and oxidative stress indicators in patients with liver and kidney deficiency OA, and is superior to local acupoint warming acupuncture.

Zeqiu et al^[60]^ designed RCT to explore the clinical efficacy of hemi-needling with filiform fire needle in the treatment of OA patients with cold-dampness obstruction in acute stage. In this study, 80 patients with acute OA of cold-dampness obstruction type were randomly divided into observation group and control group, with 40 patients in each group. The control group was treated with conventional western medicine, while the observation group was treated with hemi-needling therapy on the basis of the control group, with 7 days as a course of treatment and 2 consecutive courses of treatment. The changes of VAS score, Lequesne index score and the contents of IL-6 and TNF-α in synovial fluid were observed before and after treatment. The study found that on the basis of conventional western medicine treatment combined with the filiform fire needle half needling method in the treatment of acute OA patients with cold-dampness blockage type has a definite effect, which can effectively alleviate joint pain and improve joint function, and its mechanism may be related to the inhibition of inflammatory reaction.

## 10. Treatment of OA by acupuncture combined with medicine

Hongpeng et al^[61]^ observed the clinical efficacy of acupotomy combined with Qishao Tongbi Capsule in the treatment of KOA (cold-dampness arthralgia syndrome). According to the method of random number table, 120 patients were divided into needle-knife group, Chinese patent medicine group and combined group, 40 cases in each group. The course of treatment was 4 weeks in each group. VAS score, Lysholm score, WOMAC score and serum erythrocyte sedimentation rate, CRP, IL-1β levels were observed before and after treatment in each group. The study found that acupotomy combined with Qishao Tongbi capsule can effectively improve knee pain and activity function, regulate serum erythrocyte sedimentation rate, CRP and IL-1β levels, and has a good clinical effect on the treatment of KOA.

Renzhong et al^[62]^ designed RCT to observe the clinical efficacy of kidney-tonifying, blood-activating and collateral-dredging acupuncture combined with fumigation and washing therapy of TCM in the treatment of KOA. In this study, 76 patients with KOA were randomly divided into control group and observation group, with 38 cases in each group. The control group was treated with conventional western medicine, while the observation group was treated with TCM fumigation therapy on the basis of the control group. VAS score, WOMAC, Lequesne MG score, synovial fluid IL-1β, IL-6, TNF-α levels, and serum cyclooxygenase-2 (cyclooxygenase-2, cyclooxygenase-2) were observed before and after treatment. The levels of COX-2 and matrix metalloproteinase-3 (MMP-3) and the clinical efficacy. Conclusion Bushen Huoxue Tongluo acupuncture combined with TCM fumigation and washing therapy can significantly improve the clinical symptoms of patients with KOA, reduce pain and improve knee joint function.

Dai Li^[63]^ designed RCT to analyze the effect of Buyang Huanwu Decoction plus Erchen Decoction combined with acupuncture on IL-1, IL-6, MMP-13 and electrophysiology in patients with elbow OA secondary to mild to cubital tunnel syndrome. In this study, 104 patients with elbow OA were selected and divided into observation group and control group according to different treatment methods, with 52 cases in each group. Both groups were treated with anti-inflammatory and neurotrophic drugs, while the control group was treated with Buyang Huanwu Decoction and Erchen Decoction, and the observation group was treated with acupuncture on the basis of the control group. Both groups were treated for 4 weeks. The clinical efficacy, VAS and disabilities of arm, shoulder and hand were compared between the 2 groups before and after treatment. DASH score, IL-1, IL-6, MMP-13 levels, motor conduction velocity and sensory conduction velocity of ulnar nerve at elbow segment. Sensory conduction velocity, nerve action potentia at the elbow segment of the ulnar nerve trunk and adverse events. Conclusion Buyang Huanwu Decoction plus Erchen Decoction combined with acupuncture has a definite therapeutic effect on cubital tunnel syndrome secondary to elbow OA, which can reduce the levels of IL-1, IL-6 and MMP-13, relieve pain, promote the recovery of upper limb function, and improve neuroelectrophysiology with high safety.

## 11. Discussion

With the aggravation of the aging of Chinese society, the incidence of OA is increasing year by year, which has a serious impact on the physical, mental health and quality of life of patients, and has caused great burden to the family, economy and society, so more and more attention has been paid to the treatment of OA. TCM has a long history of research and prevention and treatment of OA, and has gradually formed rich clinical treatment experience through the summary and innovation of doctors in past Dynasties. With the progress of science and technology, the in-depth study of modern medicine, as well as the multi-level and multi-angle joint study of multi-disciplinary and TCM, the mechanism of action of TCM on OA has been continuously studied and revealed, providing more reliable and convincing experimental evidence for clinical practice, making the treatment of OA with TCM both scientific and effective.

In recent years, great progress has been made in the experimental research on the treatment of OA by TCM. Studies have shown that TCM therapy can restore the metabolic balance of articular cartilage by inhibiting chondrocyte apoptosis, promoting chondrocyte proliferation, maintaining the normal expression of matrix collagen, improving the metabolism of abnormal oxygen free radicals, and inhibiting the release of inflammatory factors. These conclusions provide a lot of scientific basis for the clinical treatment of OA with TCM.^[64]^

However, at present, there are still some deficiencies in the treatment and scientific research of OA in TCM, and the evaluation methods of drug efficacy and adverse reactions are not perfect; clinical medication is not really different from person to person, staging and typing are appropriate, some diseases have no clear staging of TCM, and the combination of treatment methods and constitution is not enough; Lack of attention to TCM decoction and nursing after medication may be one of the reasons why the efficacy of drugs can not meet expectations; most of the external treatment methods require professional operation, which is difficult for patients to complete alone, and there is a lack of professional guidance for the continuation of treatment after discharge; the operation duration, intensity and frequency of some external treatment methods have not yet formed a unified standard, and the wrong treatment methods and intensity may aggravate the condition.

The clinical and animal studies of OA in TCM are mostly focused on knee OA, and the studies on hand, foot, hip, spine and other joints are less, lacking universality and representativeness, and can not objectively evaluate the efficacy of treatment; there is no special unified standard for syndrome differentiation and typing in TCM, and different diagnosis and treatment guidelines have different syndrome types, which leads to difficulties in clinical promotion; At present, although there is a large regional epidemiological survey, there is a lack of nationwide large-scale epidemiological survey; most clinical trials lack long-term efficacy observation, such as the length of efficacy, adverse reactions, recurrence rate, etc.; in most of the existing studies, there are many subjective indicators to evaluate the clinical efficacy of OA, and there is a lack of strong objective indicators such as imaging and biomedical results; The preparation of animal models is mainly based on the “disease” model of Western medicine, and the research ideas and methods are mostly based on Western medicine, which is lack of coincidence with the theory of disease differentiation and syndrome differentiation of TCM, and the lack of perfect animal models makes it difficult to make breakthroughs in drug research and development; most of the studies fail to implement the principle of blindness, which makes the results biased.

To sum up, the research of TCM in the treatment of OA has a long way to go.

## Author contributions

**Conceptualization:** Biao Qi.

**Data curation:** Biao Qi, Zeyu Wang, Ying Cao, Haishen Zhao.

**Formal analysis:** Biao Qi.

**Funding acquisition:** Biao Qi.

**Investigation:** Biao Qi.

**Methodology:** Biao Qi.

**Project administration:** Biao Qi.

**Resources:** Biao Qi.

**Software:** Biao Qi.
